# Innovative Monitoring of Atmospheric Gaseous Hydrogen Fluoride

**DOI:** 10.1155/2016/2129053

**Published:** 2016-10-13

**Authors:** Stefano Dugheri, Alessandro Bonari, Ilenia Pompilio, Alessandro Monti, Nicola Mucci, Giulio Arcangeli

**Affiliations:** ^1^Laboratorio di Igiene e Tossicologia Industriale, Azienda Ospedaliero-Universitaria Careggi, Largo P. Palagi 1, 50100 Firenze, Italy; ^2^Dipartimento di Medicina Sperimentale e Clinica, University of Florence, Largo G.A. Brambilla 3, 50139 Firenze, Italy; ^3^Fondazione per la Ricerca e l'Innovazione, Polo Scientifico, Via Madonna del Piano 6, 50019 Sesto Fiorentino, Italy

## Abstract

Hydrogen fluoride (HF) is a basic raw material for a wide variety of industrial products, with a worldwide production capacity of more than three million metric tonnes. A novel method for determining particulate fluoride and gaseous hydrogen fluoride in air is presented herewith. Air was sampled using miniaturised 13 mm Swinnex two-stage filter holders in a medium-flow pumping system and through the absorption of particulate fluoride and HF vapours on cellulose ester filters uncoated or impregnated with sodium carbonate. Furthermore, filter desorption from the holders and the extraction of the pentafluorobenzyl ester derivative based on solid-phase microextraction were performed using an innovative robotic system installed on an* xyz* autosampler on-line with gas chromatography (GC)/mass spectrometry (MS). After generating atmospheres of a known concentration of gaseous HF, we evaluated the agreement between the results of our sampling method and those of the conventional preassembled 37 mm cassette (±8.10%; correlation coefficient: 0.90). In addition, precision (relative standard deviation for *n* = 10, 4.3%), sensitivity (0.2 *μ*g/filter), and linearity (2.0–4000 *μ*g/filter; correlation coefficient: 0.9913) were also evaluated. This procedure combines the efficiency of GC/MS systems with the high throughput (96 samples/day) and the quantitative accuracy of pentafluorobenzyl bromide on-sample derivatisation.

## 1. Introduction

Hydrogen fluoride (HF) is produced by reacting mineral fluorspar, also known as fluorite, with sulphuric acid and is used as a reactant for preparing fluorocarbons. China is the leading producer of fluorspar (59% of the world production), followed by Mexico (18%) and Mongolia (5.5%) [1]. Forty HF manufacturers worldwide are responsible for approximately 45% of the HF production [2]. In addition, aluminium fluoride production (28%), metal treatment (2%), and petroleum alkylation (2%) are other relevant manufacturing sectors where HF is widely used [3]. Hydrofluoric acid and fluorochemicals are in high demand in Asia-Pacific. Therefore, global consumption in this region is expected to increase by more than 50% by 2021, and the worldwide fluorochemical market is expected to be worth almost five billion US dollars [4]. In 2012, European HF production was around 240 000 tonnes and was valued at around 320 million euros. Furthermore, around 1000 people are directly employed in the nine HF production sites in four European countries while the estimated total number of jobs related to the fluorine industry amounts to more than 50000 [5].

For many years, the American Conference of Governmental Industrial Hygienists (ACGIH) adopted a threshold limit value- (TLV-) ceiling (C) for HF as a fluoride, with 1.053 as the conversion factor to convert fluoride to HF of 2.4 mg/m^3^ (3 ppm). In 2004, the ACGIH began providing additional information on the status of this chemical substance, and a TLV-time-weighted average (TWA) of 0.4 mg/m^3^ and TLV-C of 1.6 mg/m^3^ were published. The HF Acute Exposure Guidelines Level 1 value recommended by the United States Environmental Protection Agency (EPA) is 0.8 mg/m^3^ for 10 min of exposure. Because high concentrations of HF may cause severe acute health effects, such as respiratory damage, pulmonary oedema, hypocalcaemia, ocular irritation, and dermal burns, we conducted our investigation by using a sensitive method that allows a rapid evaluation of HF in workplaces [6–8] showing that fluoride exposure may lead to an increasing trend in cancer incidence among female workers in the aluminium industry.

The existing methods for detecting particulate fluoride and gaseous HF are based on active sampling by using 25 or 37 mm cassettes containing a mixed-cellulose ester (MCE) filter uncoated and impregnated with sodium carbonate or sodium formate and analysing it through ion chromatography (IC) or ion-specific electrode (ISE), as reported by the National Institute for Occupational Safety and Health (NIOSH) [9, 10], Occupational Safety and Health Administration (OSHA) [11], EPA [12], and Kontozova-Deutsch et al., 2011 [13]. However, these methods are not sufficiently sensitive, and they also involve many chemicals and complex extraction procedures. These problems can be resolved through solid-phase microextraction (SPME), a solvent-free technique that combines sampling, isolation, and enrichment and is suitable for gas chromatography (GC)/mass spectrometry (MS) analysis. Because of its capability to solve a wide variety of analytical sampling problems, the SPME is considered one of the major ideas that shaped analytical chemistry in the 20th century [14]. Considering the growing interest in the environmental field and the limited number of such applications, efficient, comprehensive, and reproducible methods are urgently required for both experts and nonexperts [15–19].

This study is the first to report the determination of particulate fluoride and airborne HF by applying a new open-face 13 mm Swinnex two-stage holder filter desorbed by an innovative* xyz* GC autosampler. By using a new robotic system, which allowed an on-sample SPME derivatisation in a fully automated mode, we extracted the pentafluorobenzyl ester derivative of sodium fluoride to improve the selectivity and sensitivity of the analytical method through mass spectrometry. This study performed automated assays in extremely short time periods that were characterised by a higher sensitivity power and discrimination than other routine techniques used in industrial hygiene laboratories, because of the structurally informative MS fragmentation pattern. We described our procedure and compared it with the existing methods for HF sampling. Finally, the study reports the results of a campaign of environmental monitoring conducted in an Italian company during the superbrightening of aluminium surfaces by using anodic baths with an HF base.

## 2. Materials and Methods

### 2.1. Reagents

2,3,4,5,6-Pentafluorobenzyl bromide (PFBBr; Cat. Number 101052, Aldrich Saint Louis, MO, USA), the internal standard (IS) sodium acetate (Cat. Number 71183, Fluka), sodium carbonate (Cat. Number 71345, Fluka), sodium fluoride (Cat. Number 201154, Sigma-Aldrich), and hydrofluoric acid (Cat. Number 339261, Sigma-Aldrich) were purchased from Sigma-Aldrich (Milan, Italy). Acetone (Cat. Number 8002) was purchased by J. T. Baker (Exacta-Optech Labcenter, San Prospero, Italy); 0.5 M sodium phosphate buffer (pH 6.8) was purchased from GiottoBiotech (Sesto Fiorentino, Italy).

### 2.2. Sampling Equipment

MCE filters were preloaded with aqueous sodium carbonate and preassembled in three-piece 37 mm cassettes for closed-face sampling configuration (Cat. Number 225-9001) purchased from SKC (Eighty Four, PA, USA). After sampling, each filter was transferred in a 20 mL BD luer-lock syringe (Cat. Number 302830) and manually desorbed using 10 mL of 0.5 M phosphate buffer (pH 6.8) in a 20 mL headspace (HS) vial. The mini samplers proposed in this study were configured using a polypropylene Swinnex holder (Cat. Number 225-32, SKC) with an MCE filter (diameter: 13 mm and pore size: 0.8-*μ*m; Cat. Number A080A013A, Advantec MFS, Inc., Dublin, CA, USA) impregnated with 10 mg of sodium carbonate (60 *μ*L of 0.75 M over the filter surface, allowed to dry at room temperature for 4 hours), similar to that of a prefilter assembled in an open-face 13 mm Swinnex holder (Cat. Number 225-6201, SKC). Fluorides were desorbed using 1.0 mL of 0.5 M phosphate buffer (pH 6.8) in a fully automated mode in a 20 mL HS vial, using modified Vista luer-lock 1.2 mL syringe (Cat. Number 316002, Vista Dental Products, Racine, WI, USA); the syringe was connected to a Swinnex 13 mm filter holder by using Fast Fit Assemblies (FFAs; Chromline, Prato, Italy). Moreover, to enable the positioning of the sampler inlets within the proximity of the operator's nose and mouth, we used a face-level sampling headset (Cat. Number 225-6200, SKC). The GilAir Plus portable air sampling pumps were kindly supplied by Sensydine (Recom, Genova, Italy).

### 2.3. Automation of the Analytical Procedure

The procedure was completely automated using a new Flex GC autosampler (EST Analytical, Fairfield, OHIO, USA) equipped with sample trays for 32 vials, a heated incubator shaker, barcode reader, SPME fiber-conditioning device, and Multi Tools Exchange (MTX) device patented by Chromline (Prato, Italy). The MTX device can automatically exchange tools: two 100 *μ*L syringes which are required to add a derivatising agent and IS, a FFA-SPME fiber (Supelco, Bellefonte, PA, USA), and the FFA Swinnex 13 mm filter. FFA is formed by adaptors that make the Swinnex more robust and can be identified using its barcode. Moreover, FFA can facilitate changing the Swinnex 13 mm filter holder in an automatic mode by using the MTX device. In this step, the mini samplers are transported between the 45-position tray and the vial for desorption by a new cartridge holder equipped with a plunger and magnetic system. After the analysis, desorbed Swinnex holders are moved back to the tray, and the cycle is repeated.

### 2.4. SPME On-Sample Derivatisation

The desorbed fluoride and IS acetate (50 *μ*L of 10 mg/mL 0.5 M phosphate buffer, pH 6.8) were alkylated using 20 *μ*L of a 50 *μ*L/mL PFBBr acetone solution. The mixture was heated at 80°C in a block heater for 60 min. Furthermore, a 65 *μ*m polydimethylsiloxane-divinylbenzene FFA-SPME fiber was directly immersed in the HS of the 20 mL vial for 10 min at 60°C, incubated for 5 min under continuous agitation at 500 rpm, and finally desorbed into a GC injector port for 1 min.

### 2.5. GC/MS Conditions

GC/MS analysis was performed using a Varian CP-3800 GC equipped with an electronic flow control (Varian Inc., Palo Alto, CA, USA). A fused silica methyl-deactivated capillary column (internal diameter: 10 m × 0.25 mm) was used as a guard column connected to a VF-5 ms (internal diameter: 30 m, 0.25 mm and film thickness: 0.25 *μ*m) analytical column (Cat. Number CP9013, Agilent J&W GC Columns, Agilent Technologies, Cernusco sul Naviglio, Italy). The initial column temperature was set to 40°C for 5 min and then increased at 20°C/min to 220°C, which was maintained for 1 min (total, 15 min). The injector (250°C) was set in the split mode (10 : 1), and helium at a flow rate of 1.2 mL/min was used as the carrier. Ionization was performed using an ion-trap Saturn 2200 series MS detector operating in the electron impact mode.

### 2.6. Generation of Gaseous Standard Mixtures

HF is one of the most reactive compounds to handle in the gaseous phase. Its room-temperature boiling point of 19.5°C and its chemical reactivity make HF one of the most difficult to handle compounds in the laboratory. We developed a more rapid and economical system on the basis of permeation tubes and syringe pumps, as previously reported [20, 21]. For generating air samples containing known concentrations of HF (closely representing actual air samples), we used the system proposed by Nelson, 1992 [22], with modifications. When operating, a volume corresponding to 100 *μ*L of an aqueous HF solution of a known concentration was injected using a 1 mL syringe (Cat. Number 03071250300350, Pic, Artsana, Grandate, Como, Italy) into a Teflon injector port (Chromline, Prato, Italy) at 120°C of an ATIS Adsorbent Tube Injector System (Cat. Number 28521, Supelco, Bellefonte, PA, USA) and collected in a 50 L Kinar sampling bag (Cat. Number KB3-50, Sensydine, Recom, Genoa, Italy). Furthermore, for collecting air samples to test the two analytical methods, we prepared four test atmospheres of HF each day (0.25, 1.2, 2.3, and 5.0 mg/m^3^) in deionized water. For each sampling bag, the HF concentration was determined, first with continuous sampling and analysis by using an HF electrochemical toxic gas monitor, TG-501 probe (GrayWolf Sensing Solutions, Shelton, CT, USA) equipped with a modified calibration hood (Chromline, Prato, Italy). This step was followed by the use of a preassembled three-piece 37 mm cassette for closed-face sampling configuration, aspirating at a flow rate of 2.0 L/min or two 13 mm MCE mini sampler filters (flow rate: 0.4 L/min) connected to a sampling pump. The HF air concentration (*C*_HF air_) was calculated according to the following formula:

(1) $$\begin{array}{c} C_{HF\;air}=\frac{m_{sol}}{V}, \end{array}$$

where, *C*_HF air_ is the concentration of the analyte in air (*μ*g/L), *m*_sol_ is the mass of the HF in an aqueous solution injected (*μ*g), and *V* is the volume (L) of the air in the Kinar sampling bag.

## 3. Results and Discussion

In this study, short sampling periods for the rapid assessment of brief acute exposure as well as the long-term monitoring of HF in the work places in addition to automated analysis coupled with HS-SPME have been investigated as a possible alternative to conventional methods. In previous studies, the clean-up step has often been used to extract and eliminate most of the interfering compounds from the collected air. This study aimed to develop a fully automated, rapid, sensitive, and organic solvent-free innovative procedure for monitoring atmospheric particulate fluoride and gaseous HF. Therefore, to develop a successful method, we satisfied three fundamental requisites.

### 3.1. Mini Sampler

Gaseous HF is highly soluble in water; therefore, aerosol particles may act as carriers of HF into the alveolar region of the lower respiratory tract. On the basis of a one-dimensional mass balance model, it was estimated that, under peak exposure conditions, approximately 10% of the initial gaseous HF would be transferred to the particle phase [23]. The 0.8 *μ*m pore size MCE prefilter removes particulate fluoride from the air stream before contact with the sodium carbonate-impregnated filter. Therefore, the simultaneous peak exposure to both HF and hygroscopic aerosols is essential to evaluate the occupational exposure. In the conventional IC, interference by organic particles in fluoride measurement can make this analytical technique unsuitable for measuring the TLV-C.

Limited information is available on commercial instruments that can measure short-term peak HF concentrations. Electrochemical sensors are small and convenient portable instruments; however, these sensors can have considerable cross-sensitivities to sulphur dioxide. A patented amperometric-electrochemical TA-2102 HF sensor [24] eliminates the common interfering gases. Furthermore, tuneable diode laser absorption spectroscopy in the near-infrared region was designed by Linnerud et al., 1998 [25], for HF monitoring and commercialised with a newly developed LaserGas III Portable HF Analyser [26, 27]. Limitations of these instruments include their nonportability, inability to sample particulate fluoride, and relatively low detection limits (0.1 ppm).

We proposed a mini sampler suitable for evaluating the exposure to particulate fluoride and airborne HF for comparing TLV-TWA and TLV-C. Particulate fluoride was collected into the MCE filter by using a Swinnex 13 mm open-face filter holder connected to a backup conventional Swinnex holder assembled with a 13 mm MCE filter impregnated with sodium carbonate for sampling HF vapours. Lidén and Surakka (2009) [28] developed this open-face mini sampler by using a modified Millipore (Billerica, MA, USA) Swinnex holder. The sampler has an entry nozzle of aluminium (diameter: 10 mm and length: 9 mm) and of the total length 7 mm is a polytetrafluoroethylene-sealed O-ring that protrudes out of the front of the filter holder. The sampler design indicated that it can be used for sampling the inhalable fraction of aerosol, for which 90% of the mass size distribution is below 20 *μ*m. Moreover, the backup Swinnex allowed 94% ± 8% (4 mg/m^3^; sampling time: 480 min) HF retention, in accordance with that proposed by Demange et al., 2011 [29], OSHA methods n. 21 [30], and PV2024 [31]. Desorption with 1.0 mL of 0.5 M phosphate buffer (pH 6.8) allowed a 98% recovery of fluoride from MCE filters impregnated with sodium carbonate (mean recovery of the spiked sodium fluoride).

Skaugset et al., 2013 [32], tested Respicon, 25 mm closed-face total dust cassette, and Institute of Occupational Medicine-inhalable aerosol devices comparing aerosol mass and water-soluble fluoride sampling performance. Therefore, such studies should be conducted using new samplers to comprehensively understand the difference and reproducibility of different systems in field comparisons, which substantially differ from wind-tunnel studies.

### 3.2. PFBBr Derivatisation and HS-SPME

Fluorine does not participate in chemical reactions that are adequately selective to permit its direct determination in the presence of concomitant elements [33]. Consequently, no direct or specific spectrometer, electrochemical sensor, or fluorescence methods are available for its quantitation. A significant advancement in the determination of fluoride was the development of the ISE [34]. Owing to its simplicity, the ISE was used as the NIOSH reference method until 2014, when the NIOSH Method 7906 by IC of the previous reaction with sodium carbonate was proposed. However, in the IC analysis, cosampled formate and acetate compounds in the work environment can cause a positive interference, whereas cations that form insoluble fluorides, such as Fe^3+^, Ca^2+^, and Al^3+^, can cause negative interference. Therefore, these methods detect fluoride only on the basis of its absorbance and RT and sometimes lack specificity.

Gas chromatographic analysis coupled to a mass spectrometer detector can identify analytes by using both their RTs and mass spectra; therefore, it is extremely common in examining hygiene in industries. GC/MS previous on-sample derivatisations of fluoride ions in an aqueous sample have recently been performed using triethyloxonium tetrachloroferrate(III), which is not commercially available and in a HS vial to yield fluoroethane [35]. Kage et al., 2008 [36], used PFBBr followed by liquid-liquid extraction with n-hexane; in a similar manner, we have developed a new and different approach by using on-sample derivatisation followed by HS-SPME. A study proposed three procedures for SPME derivatisation by using PFBBr: on-sample and HS or direct immersion extraction, on-fiber, and in the injector [37]. A sensitive GC method has been established for determining anions, including cyanide, formate, acetate, iodide, nitrite, nitrate, sulphide, and thiocyanate, as their volatile organic derivatives by using PFBBr [38–42]. Because the performance of SPME in determining anions after alkylation with PFBBr has not been reported, the equilibrium and kinetics of fluoride in SPME have been discussed on a theoretical basis. An effective use of the theory minimizes the number of experiments to be performed; however, the assumption of ideal conditions required by the mathematical modelling requires verification. Therefore, the constant of distribution estimated from physicochemical tables or by using the structural unit contribution method can anticipate trends in SPME analysis. Furthermore, Performs Automated Reasoning in Chemistry is a physicochemical calculator that uses computational algorithms based on the fundamental chemical structure theory to estimate a wide variety of reactivity parameters strictly from molecular structures [43]. The constant of Henry of the fluoride pentafluorobenzyl ester derivative was 5080 atm cm^3^/mol (Table 1), which was in agreement with that reported by Pacenti et al., 2008 [43], and indicated that HS-SPME is efficient for compounds with the constant of Henry higher than 34 atm cm^3^/mol analysed through GC/MS.

Regarding on-fiber and in-the-injector derivatisation, we observed that excess PFBBr causes interference in the chromatographic separation system.

### 3.3. *xyz* Axes Robotic System

In the last 10 years, miniaturisation has attracted much attention in analytical chemistry and has driven solvent and sample savings, sample enrichment, rapid sample preparation, and easier automation, resulting in the proliferation of* xyz* autosamplers. Sample preparation remains one of the more time-consuming and error-prone aspects of analytical chemistry. New sample preparation techniques are being increasingly introduced because of the considerable need for information management, the automation of sample preparation, and the integration of data management into the analytical process. Modern autosamplers and workstations possess a range of capabilities, in addition to simple liquid injection, that allow the automation of sample preparation steps traditionally performed manually. Furthermore, the flexibility of the* xyz* robotic autosampler has been useful to set up and integrate all sampling management processes and software implementation of the Flex GC autosampler. A connection with the Laboratory Information Management System (Bika Lab System) allows a user-programmable suite; therefore, customised processing steps could be easily created by the analyst. The new autosampler platform proposed in this study can automatically exchange tools (in this instance, a FFA Swinnex 13 mm filter, two 100 *μ*L syringes, and a FFA-SPME fiber) by using the MTX device. Several sample preparation steps immediately before sample injection have been automated, allowing just-in-time sample preparation.

### 3.4. Analytical Results

**Figure 1:**
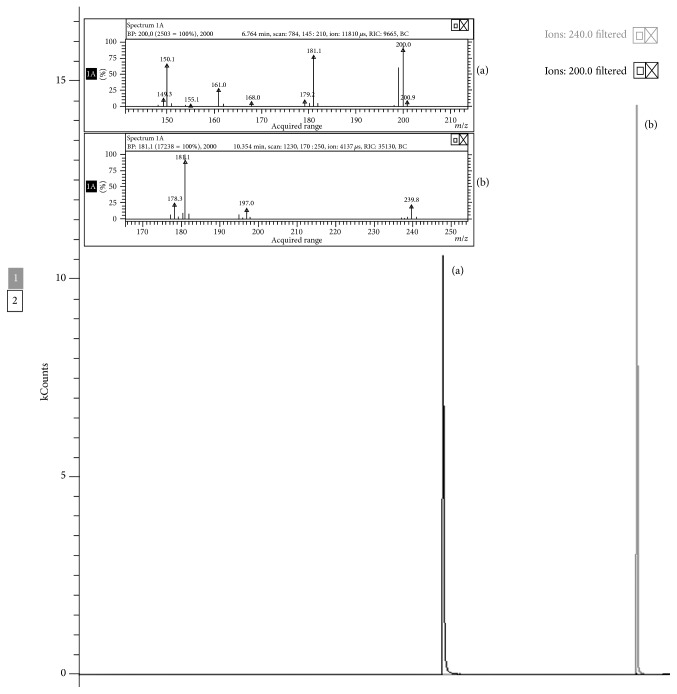
GC/MS chromatogram and EI mass spectrum of the (a) fluoride- and (b) acetate-2,3,4,5,6-pentafluorobenzyl bromide ester derivatives.

For recovery studies, the linearity range and accuracy test samples were prepared by spiking the filters with sodium fluoride. Five filters were spiked for each target concentration, and three filters were spiked only with a 0.5 M phosphate buffer (pH 6.8) solution. The precision of the GC/MS analysis of replicates (*n* = 10) of the 20.0 *μ*g/filter fluoride concentration was indicated by a relative standard deviation (4.3%). Standard solutions of sodium fluoride (1–10 mg/mL) were prepared to contain fluoride at concentrations of 2, 8, 16, 80, 160, 400, 1000, 2000, and 4000 *μ*g/filter. These samples were derivatised and extracted as described in the preceding section. The linearity from 2.0 to 4000 *μ*g/filter showed a correlation coefficient of 0.9913. Furthermore, calibration curves were constructed by plotting the peak area ratio of the base peak of the fluoride derivate at* m/z* 200 (retention time, RT 6.73 min) to the base peak of acetate (internal standard) at* m/z* 240 (RT: 10.32 min) against the fluoride concentration through MS (Figure 1). The instrumental detection limit (LOD) was calculated as [(*Y*_B_ + 3*S*_B_)*m* − 1], where *Y*_B_ is the intercept, *S*_B_ is the standard deviation, and *m* is the plot slope. The lower limit of quantification (LLOQ, corresponding to 3.3 LOD) of HF, detected as the pentafluorobenzyl ester derivative of sodium fluoride, was 0.2 *μ*g/filter and 1.0 *μ*g/filter when 1.0 and 10.0 mL of 0.5 M sodium phosphate buffer (pH 6.8) extraction solution were used, respectively. By generating standard atmospheres of a known concentration of gaseous HF, we evaluated the agreement between the results of this new sampling method (Table 2) and the conventional preassembled 37 mm cassette (±8.10%; correlation coefficient: 0.90). This method allows analysing 96 samples/day.

We applied our method in an Italian company during the superbrightening of aluminium surfaces by using anodic baths with an HF base and compared it with the conventional preassembled 37 mm cassette used simultaneously. Particulate fluoride and gaseous HF in air were determined during the dipping of downlight reflectors into industrial polyvinylidene difluoride basin (95 × 100 × 100 cm, 5% HF) equipped with an extractor hood. A favorable agreement was observed between the values (Table 3).

## 4. Conclusion

For air sampling and analysis in work environments, we have optimised an analytical method that is robust, sensitive, and simple because of the automation. The attained sensitivity permits evaluating the HF concentration with decreased sampling periods, yielding an instantaneous measurement of HF concentrations. The quality of the GC/MS approach allows an excellent resolution, even at a short analysis time, to resolve the analytes of interest from similar compounds that would interfere with the assay. Moreover, because of the new configuration autosampler *xyz*, we can provide a more desirable traceability of the sampling and full automation of the analysis.

## Figures and Tables

**Table 1:** Physical properties and partition coefficients of the fluoride-2,3,4,5,6-pentafluorobenzyl bromide ester derivative evaluated using SPARC software.

SMILES strings	CAS n.	*T*_eb_ (°C)	*D*_water_ (cm^2^/s)	*D*_air_ (cm^2^/s)	Henry's constant (atm m^3^/mol)	*K*_ow_	*P*_vap_ Log (atm)	Coefficient activity
FCC1=C(F)C(F)=C(F)C(F)=C1F	22006-43-5	148.1	7.88*∗*10^−6^	0.0643	5.08*∗*10^−3^	3.5 log	−2.15	4.6 log

**Table 2:** Performance evaluation of HF measurements from test atmospheres.

Concentration (mg/m^3^)	Mini sampler			Cassette		
	SD^1^	RSD^2^	*U* ^3^	SD^1^	RSD^2^	*U* ^3^
0.25	0.008	3.3	0.20	0.007	2.9	0.22
1.2	0.037	2.8	0.19	0.041	1.7	0.19
2.3	0.031	1.4	0.21	0.030	1.4	0.18
5.0	0.081	1.9	0.16	0.077	1.6	0.16

^1^Standard deviation.

^2^Relative standard deviation.

^3^Expanded measurement uncertainty (in accordance with UNI EN 482:1998).

**Table 3:** HF concentration (mg/m^3^) measured in an Italian company during the superbrightening of aluminium surfaces of downlight reflectors (sampling time: 15 min).

Working operation	Mini sampler		Cassette	
	Fluoride	HF	Fluoride	HF
Plant on	<0.030	0.035	<0.030	0.029
After dipping n. 1 downlight reflector	<0.030	0.063	<0.030	0.066
After dipping n. 10 downlight reflector	0.048	0.376	0.059	0.401
After dipping n. 20 downlight reflector	0.059	0.479	0.061	0.486
Plant off	<0.030	0.043	<0.030	0.049
